# Stomach antral endocrine cells in patients with irritable bowel syndrome

**DOI:** 10.3892/ijmm.2014.1887

**Published:** 2014-08-08

**Authors:** MAGDY EL-SALHY, ODD HELGE GILJA, JAN GUNNAR HATLEBAKK, TRYGVE HAUSKEN

**Affiliations:** 1 Division of Gastroenterology, Department of Medicine, Stord Helse-Fonna Hospital, Stord, Norway; 2 Division of Gastroenterology, Department of Clinical Medicine, University of Bergen, Bergen, Norway; 3 National Center for Functional Gastrointestinal Disorders, Department of Medicine, Haukeland University Hospital, Bergen, Norway; 4 National Centre for Ultrasound in Gastroenterology, Department of Medicine, Haukeland University Hospital, Bergen, Norway

**Keywords:** computer-aided image analysis, gastrin, immunohistochemistry, serotonin, somatostatin

## Abstract

To the best of our knowledge, stomach antral endocrine cells have not previously been investigated in patients with irritable bowel syndrome (IBS). Thus, in the present study, 76 patients with IBS were examined (designated as IBS-total). Diarrhoea was the predominant symptom in 26 of these patients (IBS-D), while in 21 patients, the predominant symptoms were both diarrhoea and constipation (IBS-M) and in 29 patients the predominant symptom was constipation (IBS-C). Forty-three healthy subjects were enrolled as the controls. Stomach antral biopsy samples obtained from all of the subjects were immunostained using the avidin-biotin-complex method for serotonin, gastrin, somatostatin and serotonin transporter (SERT). The immunopositive cell densities and immunoreactivity intensities were determined by computer-aided image analysis. The density of the serotonin-immunoreactive cells was significantly decreased in the IBS-M patients and increased in the IBS-C patients relative to the controls. The immunoreactivity intensity did not differ significantly between the controls and IBS-total. The density of the gastrin-immunoreactive cells was significantly greater in the IBS-D, IBS-M and IBS-C patients than in the controls. The immunoreactivity intensity of gastrin was significantly greater in the IBS-D patients than in the controls. The density of the somatostatin-immunoreactive cells cells was significantly lower in the IBS-total, IBS-D, IBS-M and IBS-C patients than in the controls. The immunoreactivity intensities of both somatostatin and SERT did not differ significantly between the controls and IBS-total. The increase in gastrin cell density and the decrease in somatostatin cell density in all IBS subtypes may cause high levels of gastric secretion, which may in turn contribute to the high incidence of dyspepsia and gastro-oesophageal reflux observed in patients with IBS.

## Introduction

The gastrointestinal tract contains several types of endocrine cells that control and regulate a number of important functions of the gastrointestinal tract, enabling it to perform its main task, the digestion and absorption of ingested nutrients (1–4). These cells are dispersed among the epithelial cells of the mucosa and have specialised microvilli that project into the lumen and function as sensors, responding to luminal stimuli, particularly nutrients, by releasing hormones that target other parts of the digestive system (5–14). There are at least 15 different populations of these cells in the gastrointestinal tract that interact in an integrated manner with each other, with the enteric nervous system, and with afferent and efferent nerve fibres of the central nervous system, in particular the autonomic nervous system (2,4).

Irritable bowel syndrome (IBS) is a common chronic functional gastrointestinal disorder that considerably reduces the quality of life and is an economic burden to both individual patients and society as a whole due to the direct costs of diagnostic tests and treatments, and the indirect costs of the low productivity of patients with IBS (4). Abnormalities of endocrine cells have been reported in the duodenum, ileum, colon and rectum of patients with IBS (15–28). However, to the best of our knowledge, the antral endocrine cells of the stomach have not been previously investigated. The antrum of the stomach contains 3 types of endocrine cells: serotonin, gastrin and somatostatin cells. Therefore, the aim of the present study was to determine whether there are any endocrine cell or serotonin transporter (SERT) abnormalities in the antrum of the stomach of patients with IBS.

## Materials and methods

### Patients and controls

Seventy-six patients with IBS, classified according to the Rome III criteria for IBS as previously described (29,30) were included in this study. Forty patients fulfilled the Rome III criteria for functional dyspepsia as well. These patients comprised 62 females and 14 males with a mean age of 32 years (range, 18–55 years). Diarrhoea was the predominant symptom in 26 of these patients (IBS-D), while in 21 patients, the predominant symptoms were both diarrhoea and constipation (IBS-M) and in 29 patients, the predominant symptom was constipation (IBS-C). All the patients (designated as IBS-total) had experienced their symptoms for many years and were unable to associate the onset of their IBS symptoms with any particular event, particularly gastrointestinal infections. They underwent a complete physical examination and were investigated by way of blood tests to exclude inflammatory, liver, endocrine or any other systemic diseases. Moreover, they underwent a colonoscopy with segmental biopsies, which revealed the presence of a normal terminal ileum, colon and rectum in all cases. None of these patients used proton pump inhibitors in the last 3 months. However, they all tried proton pump inhibitors for short periods of time without relief of symptoms.

Healthy volunteers without any gastrointestinal complaints were recruited as the controls through local announcements at Stord Hospital, Haukeland University Hospital and the University of Bergen, Norway, as well as in the local newspapers. Forty-three healthy subjects were included, of whom 15 were residents of Stord and 28 were students or hospital employees. They comprised 32 females and 11 males with a mean age of 40 years (range, 20–58 years).

The study was performed in accordance with the Declaration of Helsinki and was approved by the Regional Committee for Medical and Health Research Ethics West, Bergen, Norway. All subjects provided both oral and written consent prior to participation.

### Symptoms and quality of life assessments

The patients were asked to complete the following questionnaires: Birmingham IBS symptom questionnaire, the Short-Form (SF) Nepean Dyspepsia Index (SF-NDI) questionnaire and the Irritable Bowel Syndrome Quality Of Life (IBS-QOL) questionnaire. The control subjects were asked to complete the SF-NDI questionnaire. The Birmingham IBS symptom score questionnaire is a disease-specific score used to measure the symptoms of patients with IBS. It has been developed to be suitable for self-completion and has been found to be acceptable by patients. Its dimensions have good reliability, external validity and sensitivity (31). The questionnaire comprises 11 questions based on the frequency of IBS-related symptoms. Each question has a standard response scale with the symptoms all being measured on a 5-point Likert scale ranging from 0 (‘none of the time’) to 5 (‘all of the time’). There are 3 underlying dimensions: pain (3 items), diarrhoea (5 items) and constipation (3 items) (31). SF-NDI was primarily constructed and validated in patients with functional dyspepsia (32). Later, a Norwegian translation of the form was validated and proved to be acceptable by patients with IBS according to the Rome II criteria for IBS (33). The form is a 10-item questionnaire examining the influence of dyspepsia on the health domains in patients, namely tension/anxiety, interference with daily activities, disruption to regular eating/drinking, knowledge towards/control over disease symptoms and interference with work/study, with each subscale containing 2 items. Each item is measured by a 5-point Likert scale ranging from 1 (not at all or not applicable), 2 (a little), 3 (moderately), 4 (quite a lot) to 5 (extremely). Individual items in each subscale are aggregated to obtain a score range from 10 [lowest health-related quality of life (HRQoL) score] to 50 (highest HRQoL score) as per the original calculation formula of the developer. High scores indicate worse functioning or symptoms. The IBS-QOL questionnaire is a 34-item IBS-specific quality of life document concerning physical and psychosocial functioning as a result of IBS (34). This questionnaire includes a 5-point Likert response scale: not at all, slightly, moderately, quite a lot and extremely. IBS-QOL consists of 8 domains: dysphoria, interference with activity, body image, health worry, food avoidance, social reaction, sexual function and impact on relations. The IBS-QOL questionnaire has been validated in patients with IBS (35).

### Gastroscopy, histopathology and immunohistochemistry

Both the patients and controls underwent a standard gastroscopy, during which 5 biopsy samples were taken from the antrum from the area around the pyloric sphincter. Two biopsy samples were used in a rapid urease test for *Helicobacter pylori* (HelicotecUT Plus; Strong Biotech, Taipei, Taiwan). The remaining biopsy samples were fixed overnight in 4% buffered paraformaldehyde, embedded in paraffin and cut into 5-μm-thick sections. The sections were stained with haematoxylin-eosin, and immunostained using the avidin-biotin complex (ABC) method with a Vectastain ABC kit (Vector Laboratories, Burlingame, CA, USA). The primary antibodies used were monoclonal mouse anti-human serotonin (clone 5HT-H209, code M0758; Dako, Glostrup, Denmark), polyclonal rabbit anti-human gastrin-17 (code IR519; Dako), polyclonal rabbit anti-synthetic cyclic (1–14) somatostatin (code A0566; Dako) and monoclonal mouse anti-synthetic peptide from human SERT (code ab136607; Abcam, Cambridge, UK). The sections were incubated at room temperature for 2 h with all primary antibodies diluted to 1:100, apart from the antibody against gastrin, which was supplied in a ready-to-use form. The sections were then washed in PBS buffer (pH 7.4) and incubated with biotinylated swine anti-mouse IgG (in the case of monoclonal antibodies) or goat anti-rabbit IgG (in the case of polyclonal antibodies), both diluted to 1:200, for 30 min at room temperature. After washing the slides in PBS buffer, the sections were incubated for 30 min with peroxidase-labelled ABC diluted to 1:100, and then immersed in 3,3′-diaminobenzidine peroxidase substrate (Vector Laboratories), followed by counterstaining with haematoxylin.

### Computerised image analysis

A microscope (type BX 43; Olympus, Tokyo, Japan) equipped with built-in Koehler illumination for transmitted light, a light-intensity manager switch, a high-colour-reproductivity LED light source, a 6 V/30 W halogen bulb and a DP26 Olympus camera was used for morphometric analysis. This microscope was linked to a computer with Olympus cellSens imaging software (version 1.7). The number of immunoreactive cells, the area of epithelial cells and the immunoreactivity intensity were measured. The number of immunoreactive cells in each field was counted manually by pointing and clicking the computer mouse, and the areas of the epithelial cells were drawn manually using the computer mouse. The areas considered for quantification were those near to the muscularis mucosa. The immunoreactivity intensity in each field was measured using an automatic threshold setting. A ×40 objective was used, for which each frame (field) on the monitor represented a tissue area of 0.035 mm^2^. Measurements were made in 10 randomly selected fields for each individual. The immunostained sections from the IBS patients and the controls were coded and mixed, and measurements were made by the same person (M.E.-S.), who was blind to the identity of the individual to whom the tissue sections belonged. The data from the fields were tabulated, and the cell density of the epithelium (in cells/mm^2^) and the immunoreactivity intensity were computed.

### Statistical analysis

The gender difference and the occurrence of *Helicobacter pylori* infection between the patients and the controls was examined using Fisher’s exact test. The differences in age and quality of life measured by SF-NDI were calculated using the Mann-Whitney non-parametric test. Differences between the control, IBS-total, IBS-D, IBS-M and IBS-C groups were calculated using the Kruskal-Wallis non-parametric test with the Dunn’s post-test. The data are presented as means ± SEM values, and differences with P<0.05 were considered statistically significant.

## Results

### Patients and controls

The gender and age distributions did not differ significantly between the patients and the controls (P=0.196 and P=0.360, respectively). A total of 3 patients and 2 control subjects were positive for *Helicobacter pylori* infection, as revealed by both the urease test and histopathological examinations. The prevalence of *Helicobacter pylori* infection did not differ significantly between the patients and the controls (P=1.0). The total score of the Birmingham IBS symptom questionnaire was 21.5±0.7. The pain, diarrhoea and constipation dimensions were 7.2±0.4, 6.6±0.4 and 7.2±0.4, respectively. The total score of the SF-NDI questionnaire in the controls was 10.5±0.5 and in the IBS patients it was 26±1.2. There was a significant difference in the reduction of the quality of life according to the SF-NDI questionnaire between the controls and patients with IBS (P<0.0001). The total score of the IBS-QOL questionnaire in the patients with IBS was 72.7±1.5.

### Gastroscopy, histopathology and immunohistochemistry

The oesophagus, stomach and duodenum were macroscopically and microscopically normal in both the patients and controls. Immunoreactive cells were found in the stomach antrum of both the patients and the controls, and were either basket- or flask-shaped, sometimes with a long basal cytoplasmic process.

### Computerised image analysis

#### Serotonin

**Figure 1:**
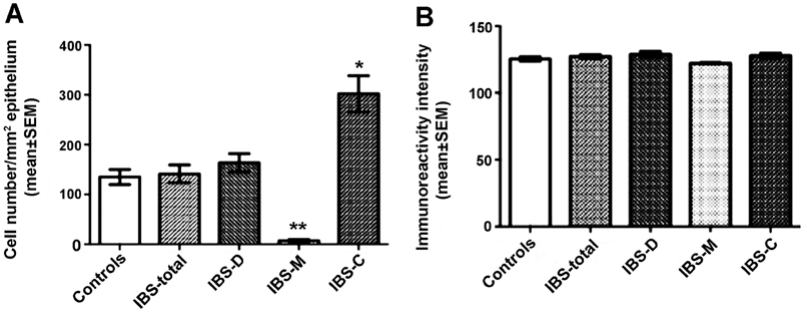
(A) Densities of serotonin-immunoreactive cells and (B) serotonin immunoreactivity intensities in the stomach antrum of the controls and IBS-total, IBS-D, IBS-M and IBS-C patients. ^*^P<0.05, ^**^P<0.01 vs. controls. IBS, irritable bowel syndrome; IBS-total, all 76 patients with IBS in this study; IBS-D, diarrhoea was the predominant symptom in 26 of these patients; IBS-M, predominant symptoms were both diarrhoea and constipation in 21 patients; IBS-C, predominant symptom was constipation in 29 patients.

**Figure 2:**
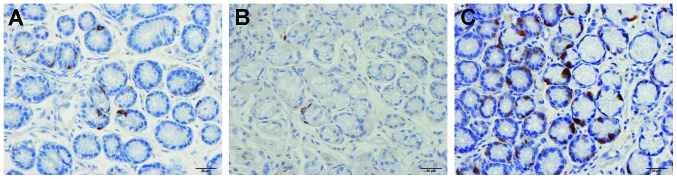
Antral serotonin-immunoreactive cells in (A) a control subject, in (B) a patient with IBS-M and in (C) a patient with IBS-C. IBS, irritable bowel syndrome; IBS-M, predominant symptoms were both diarrhoea and constipation; IBS-C, predominant symptom was constipation.

The densities of the serotonin-immunoreactive cells were 135.0±15.0, 141.3±17.9, 163.4±18.2, 7.2±2.3 and 302.3±36.3 cells/mm^2^ in the control, IBS-total, IBS-D, IBS-M and IBS-C groups, respectively. The density of the serotonin-immunoreactive cells was significantly lower in the IBS-M group and higher in the IBS-C group compared with the controls (IBS-C, P<0.05; IBS-M, P<0.01 compared to controls; Figs. 1 and 2). The immunoreactivity intensities of serotonin were 125.3±1.5, 127.1±1.3, 128.8±2.1, 122.2±0.4 and 127.8±1.8 in the control, IBS-total, IBS-D, IBS-M and IBS-C groups, respectively, with no statistically significant differences between any of these groups (P=0.2; Figs. 1 and 2).

#### Gastrin

**Figure 3:**
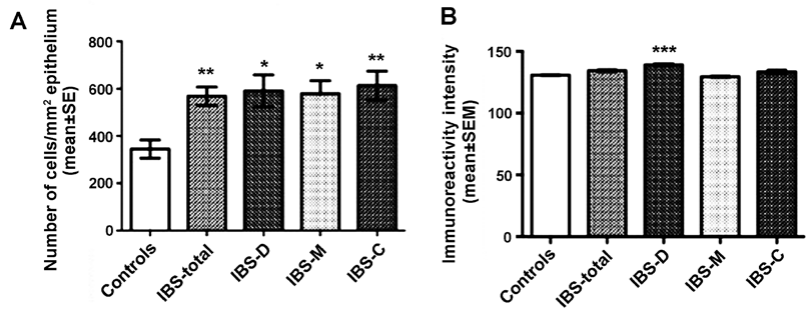
(A) Densities of gastrin-immunoreactive cells and (B) gastrin immunoreactivity intensities in the stomach antrum of the controls and IBS-total, IBS-D, IBS-M and IBS-D patients. ^*^P<0.05, ^**^P<0.01, ^***^P< 0.0001 vs. controls. IBS, irritable bowel syndrome; IBS-total, all 76 patients with IBS in this study; IBS-D, diarrhoea was the predominant symptom in 26 of these patients; IBS-M, predominant symptoms were both diarrhoea and constipation in 21 patients; IBS-C, predominant symptom was constipation in 29 patients.

**Figure 4:**
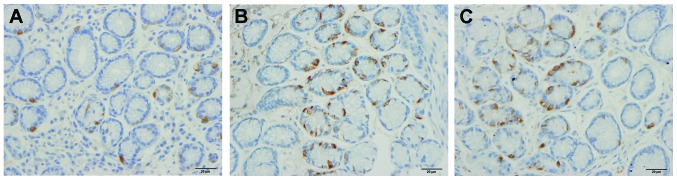
Gastrin-immunoreactive cells in the stomach antrum of (A) a control subject, (B) a patient with IBS-D and (C) a patient with IBS-C. The immunoreactivity intensity is highest in the patient with IBS-D. IBS, irritable bowel syndrome; IBS-D, diarrhoea was the predominant symptom; IBS-C, predominant symptom was constipation.

The densities of the gastrin-immunoreactive cells were 344.8±38.2, 567.9±38.9, 591.0±67.6, 579.1±54.7 and 612.9±61.1 cells/mm^2^ in the control, IBS-total, IBS-D, IBS-M and IBS-C groups, respectively. The densities of gastrin-immunoreactive cells differed significantly between the controls and the IBS-total and the IBS subgroups (P<0.0001). The density of the gastrin-immunoreactive cells differed significantly between the controls and the IBS-total, IBS-D, IBS-M and IBS-C patients (P<0.01, P<0.05, P<0.05 and P<0.01, respectively; Figs. 3 and 4). The immunoreactivity intensities of gastrin were 130.8±0.8, 134.6±1.0, 139.3±1.3, 129.7±0.9 and 133.7±1.7 in the control, IBS-total, IBS-D, IBS-M and IBS-C groups, respectively, with statistically significant differences between all groups (P=0.0001). The gastrin immunoreactivity intensity was significantly higher in the IBS-D patients than in the controls (P=0.0001; Figs. 3 and 4).

#### Somatostatin

**Figure 5:**
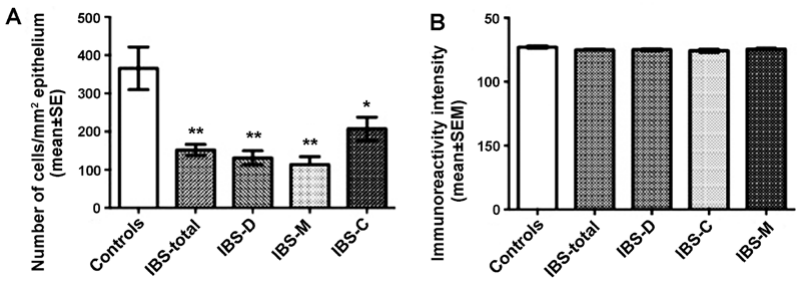
(A) Somatostatin-immunoreactive cell densities and (B) somatostatin immunoreactivity intensities in IBS-total, IBS-D, IBS-M and IBS-D patients. ^*^P<0.05, ^**^P<0.01 vs. controls. IBS, irritable bowel syndrome; IBS-total, all 76 patients with IBS in this study; IBS-D, diarrhoea was the predominant symptom in 26 of these patients; IBS-M, predominant symptoms were both diarrhoea and constipation in 21 patients; IBS-C, predominant symptom was constipation in 29 patients.

**Figure 6:**
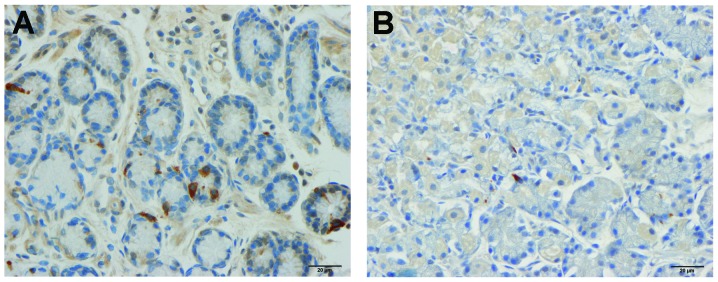
Stomach antral somatostatin-immunoreactive cells in (A) a control subject and in (B) a patient with IBS-C. IBS, irritable bowel syndrome; IBS-C, predominant symptom was constipation.

The densities of somatostatin-immunoreactive cells were 365.6±55.9, 152.0±14.6, 131.5±18.3, 113.4±21.2 and 207.3±30.5 cells/mm^2^ in the control, IBS-total, IBS-D, IBS-M and IBS-C groups, respectively. There was a statistical difference between the controls and the IBS subgroups (P=002). The density of the somatostatin-immunoreactive cells was significantly lower in the IBS-total, IBS-D, IBS-M and IBS-C patients than in the controls (P<0.01, P<0.01, P<0.01 and P<0.05, respectively). There was no significant difference in somatostatin immunoreactivity intensity between the controls (127.1±0.9) and the IBS-total (124.8±0.5), IBS-D (125.0±0.7), IBS-M (125.6±0.6) and IBS-C (124.3±1.0) patients (P=0.369; Figs. 5 and 6).

#### SERT

**Figure 7:**
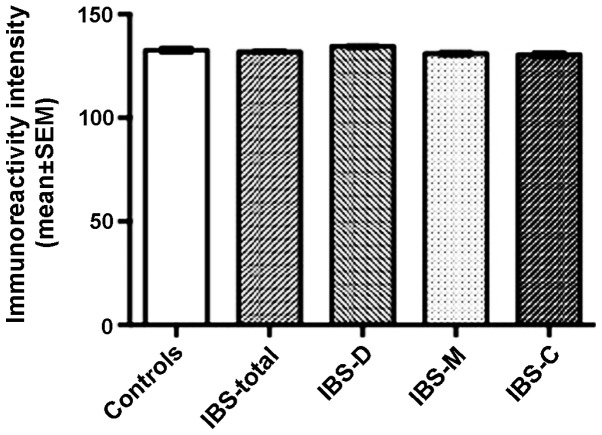
SERT immunoreactivity intensities in IBS-total, IBS-D, IBS-M and IBS-D patients. IBS, irritable bowel syndrome; IBS-total, all 76 patients with IBS in this study; IBS-D, diarrhoea was the predominant symptom in 26 of these patients; IBS-M, predominant symptoms were both diarrhoea and constipation in 21 patients; IBS-C, predominant symptom was constipation in 29 patients.

**Figure 8:**
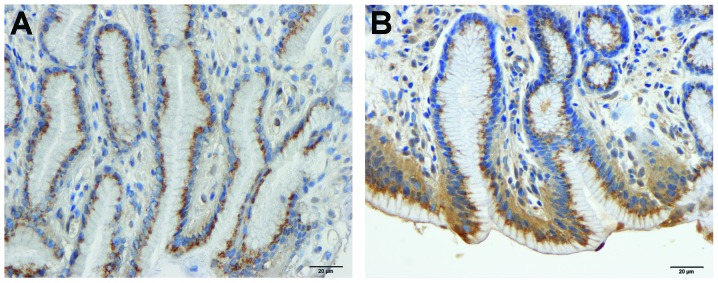
SERT immunoreactivities in the stomach antral epithelium in (A) a control subject and (B) in a patient with IBS-D. IBS, irritable bowel syndrome; IBS-D, diarrhoea was the predominant symptom.

The immunoreactivity intensities for SERT were 132.7±1.4, 132.0±0.7, 134.5±0.8, 131.1±1.2 and 130.5±1.4 in the control, IBS-total, IBS-D, IBS-M and IBS-C groups, respectively, with no significant differences between any of these groups (P=0.142; Figs. 7 and 8).

## Discussion

The patients with IBS examined in this study had moderate symptoms. These symptoms appeared, however, to considerably reduce their quality of life. Approximately 53% of these patients suffered from functional dyspepsia in addition to IBS.

The present study measured the density of endocrine cells, which are the anatomical units responsible for the production of hormones. Furthermore, the immunoreactivity intensity, which reflects the cellular hormone (secretory granules) content, and hence the summation of the cellular synthesis and release of that particular hormone was detected. The immunoreactivity intensity is a semi-quantative method measured in arbitrary units and is useful for comparing groups immunostained under the same conditions. Modern advances in the illumination used to visualise specimens on microscopes and in computer software have now made it possible to obtain reliable measurements of this parameter. The findings of the present study revealed abnormalities in all the endocrine cell types in the stomach antrum, namely the serotonin-, gastrin- and somatostatin-secreting endocrine cells. In patients with IBS, regardless of the subtype, the density of gastrin-secreting cells increased, while that of the somatostatin-secreting cells decreased.

The density of serotonin-secreting cells differed between the IBS subtypes. Whereas the density of serotonin-secreting cells was unaltered in the IBS-D patients (relative to the controls), it was decreased in IBS-M patients and increased in the IBS-C patients. Serotonin activates the submucosal sensory branch of the enteric nervous system, which conveys sensation from the gut to the central nervous system and stimulates the intrinsic primary afferent neurons that initiate peristaltic reflexes (36–39). The increased density of serotonin-secreting cells in the IBS-C patients may reflect an attempt to increase motility and initiate peristaltic reflexes in the presence of constipation. The increased serotonin levels in IBS-C patients may explain the nausea that these patients experience. However, it is difficult to ascertain why the density of serotonin-secreting cells decreased in the IBS-M patients, but not in the IBS-D patients based on a secondary effect on motility. Genetic abnormalities in SERT have been observed in patients with IBS and it has been reported that SERT levels are decreased in the large intestine and increased in the ileum of patients with IBS (20,40–46). There were no abnormalities in SERT immunoreactivity in the antrum of the patients with IBS included in the present study.

The density of gastrin-immunoreactive cells was higher in the patients with IBS (regardless of subtype) than in the controls. The increased gastrin immunoreactivity intensity in the IBS-D patients may be caused by either increased synthesis or decreased release of the hormone. On the other hand, the density of somatostatin-immunoreactive cells was lower in all the subtypes of IBS than in the healthy controls. Gastrin is the main hormonal stimulant of acid secretion in the stomach (47–51); it stimulates parietal cells both directly and indirectly by releasing histamine from enterochromaffin-like cells (47,48,51,52). Somatostatin inhibits acid secretion directly by acting on parietal cells and indirectly by inhibiting histamine and gastrin secretion (47–51). The present findings of increased gastrin and decreased somatostatin in all IBS subtypes may cause a high level of gastric acid secretion, which may account for the high incidence of dyspepsia and gastro-oesophageal reflux observed in patients with IBS (53–61).

In conclusion, the present study found abnormalities in all 3 endocrine cell types in the stomach antrum of patients with IBS. The nature of the abnormalities in the serotonin-secreting cells differed between the IBS subtypes, while all IBS subtypes had a high density of gastrin-immunoreactive cells and a low density of somatostatin-immunoreactive cells. Since gastrin is the main stimulator and somatostatin is the principal inhibitor of gastric acid secretion, a high gastric secretion is to be expected in patients with IBS. These findings may explain the overlap of IBS with dyspepsia and gastro-oesophageal reflux.

IBS is considered to be a large intestinal disorder and consequently the colonic and rectal endocrine cells have been the subjects of several studies to elucidate a possible role of these cells in the pathophysiology of IBS. Thus, colonic serotonin and PYY cell densities have been found to be low in both IBS-D and IBS-C patients (18). In the rectum of patients with sporadic (non-specific) IBS, the densities of PYY and enteroglucagon cells have been shown to be significantly lower and those of somatostatin-secreting cells to be significantly higher in both IBS-D and IBS-C patients compared to the controls, whereas the density of serotonin-secreting cells in these patients did not differ from that in the healthy controls (19,20). Rectal serotonin and PYY cell densities in post-infectious IBS have been reported to be elevated (22,24,26,62,63). Recently, however, abnormalities in the endocrine cells in the stomach and duodenum have been reported in patients with IB (15–28). The density of ghrelin cells in the oxyntic mucosa of the stomach has been shown to be lower in IBS-C and higher in IBS-D patients than in healthy controls (15). In the duodenum, the densities of GIP- and somatostatin-secreting cells have been shown to be decreased in both IBS-D and IBS-C patients (17). The densities of duodenal secretin and cholecystokinin (CCK) cells are decreased in IBS-D patients but unaltered in IBS-C patients (17). The duodenal serotonin cells are not affected in both IBS-D and IBS-C patients (17). Post-infectious IBS has been found to be associated with increased numbers of duodenal CCK cells but decreased numbers of serotonin cells (16). The present observation of abnormalities in the antral endocrine supports the suggestion that the endocrine cells are abnormal in all the segments of the gastrointestinal tract and IBS is a disorder that is not restricted to the large intestine. The present findings emphasise the role of gut endocrine cells in the pathophysiology of IBS. Moreover, they lend support to the assumption that abnormalities in gastrointestinal cells can explain the dysmotility, abdominal visceral hypersensitivity and abnormal gut secretion observed in patients with IBS (64,65).
